# Barbarians at the gate

**DOI:** 10.1007/s12471-012-0278-6

**Published:** 2012-04-03

**Authors:** R. J. G. Peters

**Affiliations:** Department of Cardiology, Academic Medical Center, Amsterdam, the Netherlands

The paper by Liew et al. in this issue of the Journal confirms other observations on the consequences of switching from branded to generic statins [1]. The common pattern observed is that the staggering costs of lipid-lowering therapy lead to national guidelines and policies that recommend or even dictate switching to generic preparations. This is associated, on average, with a reduction in pharmacological potency and an increase in low-density lipoprotein (LDL) plasma levels. The net effect is a reduction in costs that comes with a loss of preventive efficacy. However, this is not an inevitable consequence of these policies. Most include the possibility to switch back to branded statins if treatment targets are not reached with generic variants.

The proportion of the observed effects is obviously dependent on the population, the drugs involved and the methods and assumptions that were selected for the analysis. It is important to note that the calculations are generally based on published observations of cardiovascular event rates during 5 or 10 year follow-up [2]. The impact on lifetime risk may be significantly greater, both in relative and absolute event rates [3].

For a balanced view on the consequences of these observations, it may be appropriate to distinguish primary from secondary prevention since the effects of switching are likely greater in secondary prevention, where absolute risks of cardiovascular events are generally greater. This distinction could not be made in the present study on pharmacy data. Similarly, no data are available on the selection patterns that led to the 15% of all statin users in whom a switch was apparently considered appropriate by their physician. Potentially, this could have occurred more frequently in lower risk patients or in those who had LDL levels well below the recommended target plasma LDL level (2.5 mmol/l). In this case, the impact of switching may be smaller than was now calculated.

Irrespective of the average impact on a population, physicians who follow these guidelines need to carefully consider the consequences for their individual patient. If a decision is made to switch to a generic preparation, the new agent and its dose should at least be equivalent to the drug that is stopped and the LDL plasma level should be checked against previous values and against guideline-recommended target levels.

Interestingly, the Pfizer patent on atorvastatin has now expired in Europe and as of 19 March 2012, generic atorvastatin has been admitted to the Dutch market. It is available from Ranbaxy and from Pfizer. It remains to be established how many patients will now be switched back to atorvastatin.

Important as these observations are for lipid-lowering therapy, they signal a development that may have consequences far beyond cardiovascular prevention. Written primarily with the intention to improve the quality of care, guidelines in medicine are increasingly used as a basis for legal, financial and licensing policies. The 2006 Dutch guideline on Cardiovascular Risk Management is a good example, where professional recommendations formed the basis for legislation on the reimbursement of lipid-lowering therapy. This step reduced physicians’ freedom to select drug therapy and in fact reduced professional autonomy. The subsequent reductions in preventive therapy, as described in the paper by Liew et al., represent a serious, unintended side effect of the guideline [1].

This ‘external’ use of our guidelines has important consequences for professional societies that issue guidelines. External use should be routinely anticipated. This requires explicit attention at multiple levels, ranging from the selection of members for the guideline working group to the wording of the final document. At each level, an inherent conflict needs to be resolved between the responsibility of a physician for the individual patient and our collective responsibility for affordable health care.
